# The State Medical Society of Pennsylvania

**Published:** 1859-05

**Authors:** 


THE STATE MEDICAL SOCIETY OF PENNSYLVANIA will convene in this city on the second Wednesday in June. A full attendance is expected.

